# HAS THE AGING OF BRAZILIANS IMPACTED THE OCCURRENCE OF OSTEO-CARTILAGINOUS NEOPLASMS?

**DOI:** 10.1590/1413-785220243201e268544

**Published:** 2024-05-06

**Authors:** Marcelo Tomio Kohara, Gustavo Ferrareto Pires, André Marson Sanches, Rodrigo Pereira Amarante, Gabriela Caponero de Brito, Fernando Adami

**Affiliations:** 1. Centro Universitario Faculdade de Medicina do ABC, Disciplina de Ortopedia e Traumatologia, Grupo de Oncologia Ortopedica, Santo Andre, SP, Brazil.; 2. Centro Universitario Faculdade de Medicina do ABC, Laboratorio de Epidemiologia e Analise de Dados, Santo Andre, SP, Brazil.

**Keywords:** Bone Neoplasms, Joint, Cartilage, Aging, Epidemiology, Brazil, Neoplasias Ósseas, Articulação, Cartilagem, Envelhecimento, Epidemiologia, Brasil

## Abstract

Cancer cases and survival have increased significantly in recent decades. Objective: In this study, we sought to evidence whether bone, joint and cartilage neoplasms are increasing with the aging of the Brazilian population, using the analysis of the DATASUS and IBGE databases, between 1979 and 2020. Methods: We compared the means and the proportion of death in Brazil, to observe the confidence interval overlaps, separated by region. Comparison between genders, age group, death and specific rate were compared via proportion tests and the trend was investigated via time series analysis. Results: Through the analyses, we can see that there is an increasing trend of cases, about 2.5 times in the period. Separated by region, the Southeast stands out, with a number of deaths about 6 times higher than other regions. Conclusion: Metastatic carcinoma is the most common neoplasm treated by orthopedic surgeons, and it is essential to adapt to this future projection, with cases of pathological bone involvement resulting from metastatic carcinomas, increasingly present in the routine of orthopedic surgeons. ***Level of Evidence IV, Cross-Sectional Observational Study.***

## RESUMO

*Os casos oncológicos e a sobrevida aumentaram expressivamente nas últimas décadas. Objetivo: Investigar se as neoplasias de osso, articulação e cartilagem estão aumentando com o envelhecimento da população brasileira, por meio da análise das bases de dado do Sistema Único de Saúde (DATASUS) e do Instituto Brasileiro de Geografia e Estatística (IBGE), entre os anos de 1979 e 2020. Métodos: Realizamos a comparação de médias e de proporção de morte entre as regiões do Brasil para observar as sobreposições de intervalo de confiança em cada região. A comparação entre sexo, faixa etária, óbito e taxa específica foi feita via testes de proporção, e a tendência foi investigada via análise de séries temporais. Resultados: Foi evidenciada uma tendência crescente dos casos de neoplasia, com um aumento de cerca de 2,5 vezes no período analisado. Entre as regiões do Brasil, destacou-se o Sudeste, com número de mortes cerca de seis vezes maior que outras regiões. Conclusão: O carcinoma metastático é a neoplasia mais comum tratada pelos cirurgiões ortopédicos, e é fundamental nos adequarmos a essa projeção futura, com casos de comprometimento ósseo patológicos decorrentes de carcinomas metastáticos cada vez mais presentes na rotina do cirurgião ortopédico.* ***Nível de Evidência IV, Estudo Observacional Transversal.***

## INTRODUCTION

 Cancer is the second leading cause of death in the world and current estimates on the impact of this diagnosis are necessary for planning its control. ^1^^,^^2^ The Global Burden of Disease (GBD) methodology estimated that in 2015 there were 17.5 million cases of cancer with 8.7 million deaths. Cancer incidence has increased by 33% between 2005 and 2015, of which 12.6% were due to population growth and 16.4% due to population aging. ^1^ Metastatic carcinoma is the most common neoplasm treated by orthopedic surgeons ^3^^,^^4^ and there is evidence that 50% to 80% of carcinoma patients have bone metastases at the time of death. ^5^^,^^6^ Groups C40 and C41 from chapter II of the International Classification of Diseases-Tenth Revision (ICD-10) comprises malignant neoplasms of the bone and articular cartilage of limbs (C40) and of unspecified locations (C41), using the international nomenclature of diseases of the World Health Organization (WHO). This group includes primary diagnoses (which were rarer) of malignant bone disease, such as osteosarcomas, chondrosarcomas, fibrosarcomas and Ewing’s sarcomas; primary chondral malignant diagnoses, such as chondrosarcoma and, finally, diagnoses of metastatic carcinomas with bone involvement, such as bone metastases resulting from cancer of the breast, prostate, lung, kidney, thyroid, gastrointestinal tract, skin, and others. Today, the demographic projections released by the Population Division of the United Nations (UN) (2019 issue) make it clear that the process of population aging is moving at great strides in the world and at a much greater pace in Brazil. ^7^ The base of the population pyramid narrowed, while the remaining age groups widened. Thus, the process of population aging gives greater weight to the oncological diseases in the number and proportion of deaths in Brazil; in particular, the understanding of the spectrum of bone metastases is important to plan networks of solutions and treatments for bone diseases occurring in distant sites. In this work, we seek to demonstrate whether neoplasms of bone, joint and cartilage (ICD10: C40/C41) are increasing with the aging of the Brazilian population, using the analysis of the DATASUS database and the Brazilian Institute of Geography and Statistics (IBGE), between 1979 and 2020. As part of the epidemiological transition, the incidence of cancer is expected to increase in the future, further straining limited health resources. The challenge of achieving the proper allocation of resources for cancer prevention, early diagnosis, and curative and palliative care requires detailed knowledge ^8^^-^^10^ of the local burden of bone cancer, as it represents an oncological disease in an advanced stage. The objective of this study is to evaluate in Brazil and its regions the epidemiological profile of deaths, mean death and proportions of death resulting from neoplasms in the bone, cartilage or joint, in the period from 1979 to 2020, using secondary sources available on the public platforms DATASUS and IBGE. 

## MATERIALS AND METHODS

 Our open, publicly available research source was DATASUS, via its open website, in the highlighted section for Health Information (TABNET). We filtered for Vital statistics, Cancer (INCA site) so we could have access to its Atlas of Cancer Mortality. ^11^ The selected period was the most comprehensive available, from 1979 to 2020, narrowing the selection towards Brazil and its regions (North, Northeast, South, Southeast and Center-West). In each determined research selection, the filter was chosen for both sexes (male and female) and separated by topography by fixed grouping (C40 and C41), that is, neoplasms of bones, joint and articular cartilage. We compared the means and proportion of death between the regions of Brazil, using the following methods: Analysis of Variance (ANOVA) ^12^ to observe the confidence interval overlaps and differences between each region. Statistical comparison between genders and by age group, by death and specific rate was performed using chi-square ratio tests. The increasing trend was investigated via time series analysis by the Exponential Smoothing and Forecasting method. ^13^^-^^15^ In all the techniques used (Anova, t-test and time series analysis by Exponential Smoothing and Forecasting), all assumptions of normality and homogeneity of variances were checked before performing such analyses. 

## RESULTS

**Figure 1.**
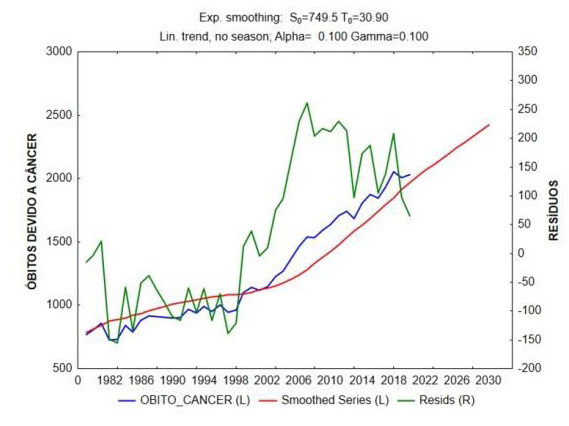
Time trend analysis and projection of deaths over the years.

**Figure 2.**
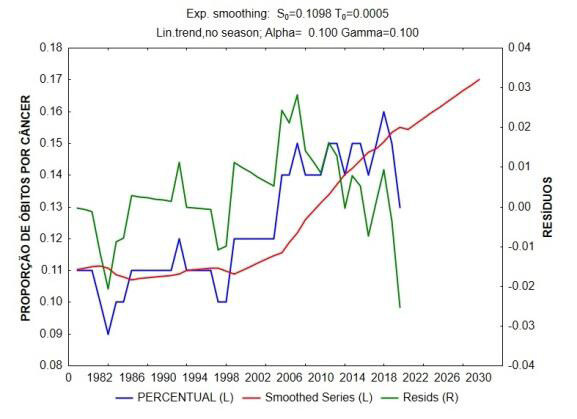
Time trend analysis and projection of the proportions of deaths due to cancer over the years.

 Through the analysis of time series by the Exponential Smoothing and Forecasting method, we can observe that there is a growing linear trend in the increase in the number of deaths due to bone, cartilage and joint cancer over the years in Brazil; and that, from 1982 to 2020, this number increased 2.5 times. There is also an increasing trend for the next 10 years (Figures 1 and 2 ). 

Time series analysis by the Exponential Smoothing and Forecasting method. In blue: the data series. In red: the smoothed series. In green: the residuals (difference between the actual and adjusted series). There is a vertiginous growing linear trend of the increase in the number of deaths due to cancer over the years in Brazil, and from 1982 to 2020, this number increased 2.5 times; the trend for the next 10 years is also increasing (trend line in red).

Time series analysis by the Exponential Smoothing and Forecasting method. In blue: the data series. In red: the smoothed series. In green: the residuals (difference between the actual and adjusted series). There is a vertiginous increasing linear trend of the increase in the proportion due to cancer over the years in Brazil.

**Figure 3.**
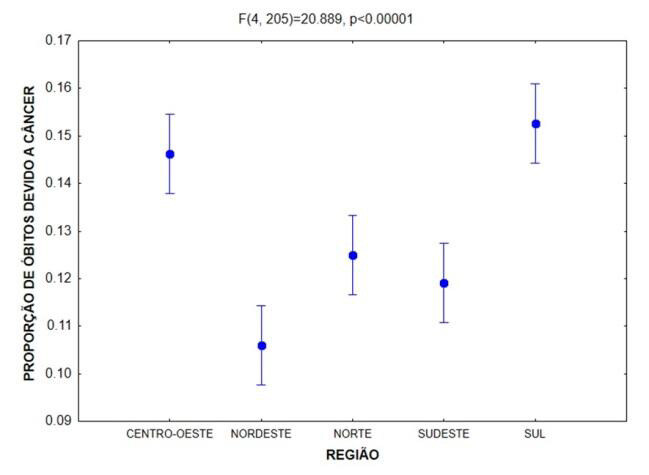
Analysis of variance (ANOVA) comparing the means of death due to cancer between regions of Brazil.

 In terms of proportion, there is a vertiginous increasing linear trend of growing proportion due to neoplasms of ICD C40 and C41 in Brazil. The ANOVA technique, comparing the number of deaths due to cancer between the regions of Brazil ( Table 1 ), showed significant statistical differences (p < 0.001) ( Figure 3 ). 

 By overlapping confidence intervals, we can see that the Southeast Region has a much higher number of deaths than the others (p < 0.0001), six times higher than the Center-West and North regions. The Northeast and South regions have about half the mortality of the Southeast region (p < 0.0001) and triple that of the Center-West and North regions (p < 0.0001). When comparing proportion of deaths, the overlap of confidence intervals shows that the South and Center-West regions have a higher proportion than the Northeast, North and Southeast (p < 0.001), but not differing among each other (p = 0.819). For rate, there are no statistically significant differences when comparing male and female patients in different age groups ( Table 2 ). 

**Table 1.** Comparison of cancer death averages between regions of Brazil, evaluated in the period from 1979 to 2020.

**REGION**	**DEATH TO CANCER Mean**	**DEATH TO CANCER Standard error**	**DEATH TO CANCER -95.00%**	**DEATH TO CANCER +95.00%**
CENTER-WEST	87.12	5.726	75.55	98.68
NORTHEAST	285.40	25.544	233.82	336.99
NORTH	69.43	6.178	56.95	81.90
SOUTHEAST	572.00	21.321	528.94	615.06
SOUTH	236.90	8.027	220.69	253.12

There are significant statistical differences (p < 0.0001). By overlapping confidence intervals, we can see that the South and Center-West regions have a higher proportion than the Northeast, North and Southeast (p < 0.001), but do not differ among each other (p = 0.819). The North and Southeast have a higher proportion than the Northeast (p < 0.01).

**Table 2.** Statistical comparison between the sexes, according to age group, deaths, and rates.

	**Male**		**Female**		**p**	
**Age range**	**Death**	**Specific rate**	**Death**	**Specific rate**	**p death**	**p rate**
0 to 4	189	0.06	232	0.07	0.03	0.977
5 to 9	400	0.12	423	0.13	0.422	0.984
10 to 14	1.246	0.36	23.130	0.36	< 0.001	1
15 to 19	2.772	0.81	1.614	0.48	< 0.001	0.774
20 to 29	3.008	0.49	1.564	0.25	< 0.001	0.782
30 to 39	1.753	0.35	1.296	0.25	< 0.001	0.897
40 to 49	2.767	0.71	2.023	0.49	< 0.001	0.840
50 to 59	4.903	1.77	3.175	1.05	< 0.001	0.668
60 to 69	5.953	3.4	3.883	1.94	< 0.001	0.527
70 to 79	4.929	5.55	3.697	3.32	< 0.01	0.454
80 or more	2.585	7.91	2.778	5.48	< 0.001	0.506
Age ignored	65	3.83	36	2.08	< 0.001	0.471
Total	30.570	-	21.951	-		

**Figure 4.**
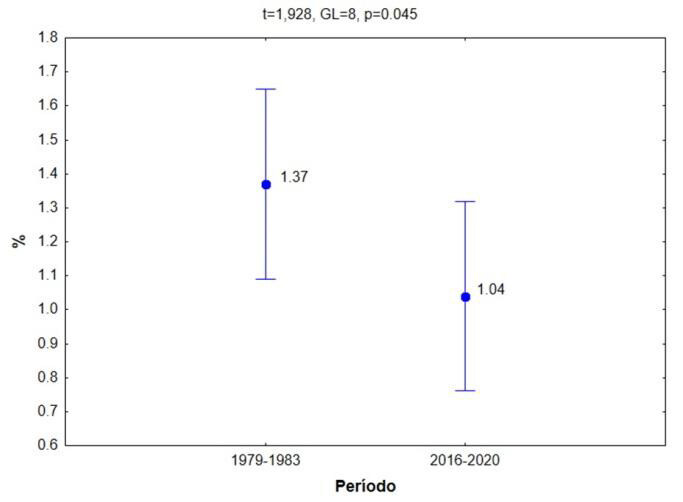
Comparison according to primary location (C40-C41) between the periods 1979-1983 and 2016-2020.

 When comparing the two broadest possible periods with the data, between 1979-1983 and 2016-2020, with the comparative t-test, the percentage distribution of total deaths from cancer of bones, joints and articular cartilage presents significant statistical differences (p = 0.045), with a lower percentage in the most recent period (2016-2020) ( Table 3 ; Figure 4 ). 

**Table 3.** Table comparison of percentage distribution of total deaths from cancer of bones, cartilage, and joints.

**Period**	**% Mean**	**% Standard error**	**% -95.00%**	**% +95.00%**
1979-1983	1.370	0.111	1.062	1.678
2016-2020	1.040	0.130	0.678	1.402

T-test comparing the percentage distribution of total deaths from cancer of bones, joint and articular cartilage. There are significant statistical differences (p = 0.045), with the most recent period (2016-2020) having a lower percentage.

## DISCUSSION

In 2015, cancer was the cause of more than 8.7 million deaths worldwide and was the second leading cause of death behind cardiovascular reasons. In Brazil, the population aging process has increased life expectancy, and the demographic projections released by the UN Population Division (2019 issue) make it clear that the population aging process is accelerating in Brazil. As a result, the country has seen an increase in cancer cases and has faced the challenge of assisting and treating this demand. By studying country-wide data sources, such as DATASUS and TABNET, for a broad period (1979 to 2020), we were able to separate a topography by fixed grouping (C40, C41: bones, joint and articular cartilage). As such, we achieved a visualization of an epidemiological sampling of a progressive increase in the number of cases, both in raw terms and proportional to the period.

 By associating the data in the time series by the Exponential Smoothing and Forecasting method, we observed a vertiginous increasing linear trend of the increase in the number of deaths due to bone, cartilage, and joint cancer over the years in Brazil; from 1982 to 2020, this number increased 2.5 times, and the trend for the next 10 years is growing. We believe that the reason for this trend is aligned with the country’s aging process. The data is compatible with the predictions determined in global oncological clinical reviews, such as the one developed by the Global Burden of Disease Cancer Collaboration ^1^ and it supports the expectation that we will have to deal with more frequent cases of bone metastases and chronic pain due to bone frailty as well as create increasingly effective solutions for these critical state patients. 

 Understanding how metastases settle into healthy tissue, evade defense systems, and continue to progress seems to be the key to treating metastatic carcinoma. These data points are fundamental for preparation and anticipation, in terms of treatment ^14^ , of a new reality in Brazilian oncology, which is developing steadily. 

 According to the database from the United States, ^15^^,^^16^ 5.1% were diagnosed with bone metastasis, which equates to roughly 18.8 per 100,000 bone metastasis diagnoses in the U.S., yearly (2010-2015). For adults, lungs are the most common primary site for cancer, followed by prostate and breast (2015 rates: 3.19 and 2.38 per 100,000, respectively). For patients under the age of 20, endocrine cancers and soft tissue sarcomas are the most common primary site. The data show an increased incidence for prostate cancers in the period. In this study, research was focused on ICD 10 (C40, C41), which includes primary bone tumors (rare) and tumors with metastatic bone involvement, without separation by age. Our perspective on the data is that the increase in cases in the country is in line with the data of this study and, in both cases, being driven by the diagnosis of metastatic bone cancer and not by osteosarcoma, Ewing’s sarcoma and other primary sites. We believe that it would be ideal to cross information with databases from the United States and the world; however, the public Brazilian database does not include the specific diagnosis of bone disease but rather groups (ICD 10). Thus, crossing of data of the same nature is currently difficult to perform. This “classification and grouping” system is fundamentally different to those of other countries. In the study by Sugiyama et al., ^17^ the cases of identification code C40 and C41(topography) were associated with the International Classification of Diseases for Oncology (ICD-O-3), and histological types were classified according to the 2013 World Health Organization system. In this description, among malignant bone tumors, the most frequent tumor types were osteogenic tumors (201; 39.7%) and chondrogenic tumors (135; 26.7%); these data are in line with previous studies, ^18^ (without inclusion of bone metastatic involvement, however), and there was no diagnosis of evolution trend (projection) in the number of cases specific to the disease. 

## CONCLUSION

In this work, we depict a vertiginous increasing linear trend of the increase in the number of deaths due to cancer in bones, cartilage and joints over the years in Brazil. From 1982 to 2020, this number increased 2.5 times, with an increasing trend expected for the next 10 years. In terms of proportion, the increase of deaths due to neoplasms with ICD C40 and C41 in Brazil is also prevalent, and this follows the enlargement of the middle and upper portion of an age pyramid of an aging country. For orthopedic professionals, it is essential to adapt to this new reality with typical cases of epidemiology of patients over 50 years, such as pathological fractures resulting from metastatic carcinomas, increasingly present and creating needs for large therapeutic options in critical patients with little functional reserve.
